# ECDC’s latest publications

**DOI:** 10.2807/1560-7917.ES.2016.21.31.30306

**Published:** 2016-08-04

**Authors:** Eurosurveillance editorial team

**Affiliations:** 1 European Centre for Disease Prevention and Control (ECDC), Stockholm, Sweden

## Rapid risk assessments

Date of publication: 14 July 2016

### 

#### Outbreaks of yellow fever in Angola, Democratic Republic of Congo and Uganda 

http://ecdc.europa.eu/en/publications/_layouts/forms/Publication_DispForm.aspx?List=4f55ad51-4aed-4d32-b960-af70113dbb90&ID=1526

Date of publication: 12 July 2016

#### Zika virus disease epidemic, seventh update

http://ecdc.europa.eu/en/publications/_layouts/forms/Publication_DispForm.aspx?List=4f55ad51-4aed-4d32-b960-af70113dbb90&ID=1525

## Scientific advice

Date of publication: 15 July 2016

### 

#### Zika virus and safety of substances of human origin – A guide for preparedness activities in Europe

http://ecdc.europa.eu/en/publications/_layouts/forms/Publication_DispForm.aspx?List=4f55ad51-4aed-4d32-b960-af70113dbb90&ID=1527

## Technical reports

Date of publication: 28 July 2016

### 

#### Hepatitis A virus in the EU/EEA, 1975–2014 

http://ecdc.europa.eu/en/publications/_layouts/forms/Publication_DispForm.aspx?List=4f55ad51-4aed-4d32-b960-af70113dbb90&ID=1542

Date of publication: 27 July 2016

#### Epidemiological assessment of hepatitis B and C among migrants in the EU/EEA 

http://ecdc.europa.eu/en/publications/_layouts/forms/Publication_DispForm.aspx?List=4f55ad51-4aed-4d32-b960-af70113dbb90&ID=1540

Date of publication: 26 July 2016

#### Public consultation: Proposals for draft EU guidelines on the prudent use of antimicrobials in human medicine 

http://ecdc.europa.eu/en/publications/_layouts/forms/Publication_DispForm.aspx?List=4f55ad51-4aed-4d32-b960-af70113dbb90&ID=1539

Date of publication: 15 July 2016

#### Seasonal influenza vaccination and antiviral use in Europe 

http://ecdc.europa.eu/en/publications/_layouts/forms/Publication_DispForm.aspx?List=4f55ad51-4aed-4d32-b960-af70113dbb90&ID=1528

## Mission report

Date of publication: 05 July 2016

### 

#### Assessing the yellow fever outbreak in Angola

http://ecdc.europa.eu/en/publications/_layouts/forms/Publication_DispForm.aspx?List=4f55ad51-4aed-4d32-b960-af70113dbb90&ID=1522

## Surveillance 

Date of publication: 18 July 2016

### 

#### Influenza virus characterisation, Summary Europe, June 2016

http://ecdc.europa.eu/en/publications/_layouts/forms/Publication_DispForm.aspx?List=4f55ad51-4aed-4d32-b960-af70113dbb90&ID=1530

Date of publication: every Friday

#### ECDC communicable disease threats report

http://ecdc.europa.eu/cdtr

